# Metamizole outperforms meloxicam in sepsis: insights on analgesics, survival and immunomodulation in the peritoneal contamination and infection sepsis model

**DOI:** 10.3389/fimmu.2024.1432307

**Published:** 2024-08-30

**Authors:** Na Liu, Mitali Sonawane, Oliver Sommerfeld, Carl-Magnus Svensson, Marc Thilo Figge, Reinhard Bauer, Sabine Juliane Bischoff, Michael Bauer, Marcin Filip Osuchowski, Adrian Tibor Press

**Affiliations:** ^1^ Department of Anesthesiology and Intensive Care Medicine, Jena University Hospital, Jena, Germany; ^2^ Applied Systems Biology, Leibniz Institute for Natural Product Research and Infection Biology-Hans Knöll Institute, Jena, Germany; ^3^ Center for Sepsis Control and Care, Jena University Hospital, Jena, Germany; ^4^ Institute of Microbiology, Faculty of Biological Sciences, Friedrich Schiller University, Jena, Germany; ^5^ Friedrich-Schiller-University Jena, Institute of Molecular Cell Biology, Jena University Hospital, Jena, Germany; ^6^ Jena University Hospital, Animal Welfare Office, Jena, Germany; ^7^ Ludwig Boltzmann Institute for Experimental and Clinical Traumatology in the AUVA Research Center, Vienna, Austria; ^8^ Medical Faculty, Friedrich-Schiller-University Jena, Jena, Germany

**Keywords:** sepsis, NSAID analgesic, clinical severity, immunophenotype, CXCR2

## Abstract

**Background:**

Limited availability and side effects of opioids have led to an increased use of non-opioid analgesia in animal disease models. However, by affecting the immune-inflammatory reactions, analgesia may disrupt the resolution of the host inflammation and modulate the survival in septic animals. This study used a clinically relevant sepsis mouse model of peritoneal contamination and infection (PCI) to investigate the antinociceptive and anti-inflammatory properties of two non-opioid analgesics.

**Methods:**

Adult C57BL/6J mice were intraperitoneally injected with a human feces suspension and received either no analgesics (Non-A), Meloxicam, or Metamizole orally. The mice were monitored for pain and illness. Mortality was assessed at 7 days post-PCI. A separate group of mice was sacrificed 24 hours after infection. Blood, peritoneal lavage fluid (PLF), liver, and spleen were harvested for pathogen load quantification via qPCR, macrophage phenotyping, neutrophil infiltration/activation, and systemic/tissue cytokine release by flow cytometry.

**Results:**

Meloxicam but not Metamizole reduced the mortality of septic mice by 31% on day 7 compared to the Non-A group. Both analgesics effectively alleviated pain but did not affect illness severity, body weight, and temperature. Meloxicam quadrupled the bacterial burden in the blood and PLF. In high IL-6 responders, Meloxicam treatment was associated with reduced circulating IL-10 and IL-1β compared to the Non-A septic group. In low IL-6 responders, Meloxicam increased circulating MCP-1 levels and decreased PGE2 levels compared to Non-A septic mice. Notably, Meloxicam reduced spleen neutrophil infiltration by 20% compared to two other sepsis groups.

**Conclusion:**

Metamizole and Meloxicam effectively relieved pain and increased the animals’ basal activity in the PCI sepsis model. Meloxicam prolonged survival yet triggered maladaptive responses due to its immunosuppressive features that decreased tissue bacterial clearance during sepsis. In contrast, Metamizole constitutes a safe and effective non-opioid alternative for analgesic control in the non-surgical PCI sepsis model.

## 1 Introduction

Sepsis, characterized by a dysregulated immune response to infection, manifests as life-threatening multiple organ failure caused by excessive or persistent pro-/anti-inflammatory reactions, immunosuppression, or combinations thereof (1, 2). Despite treatments focusing on infection clearance and restoring immune homeostasis, sepsis remains the leading cause of death in intensive care (3–6). To investigate sepsis pathophysiology and potential treatments, mouse models such as non-surgical peritoneal contamination and infection (PCI) (7) and surgical cecal ligation and puncture (CLP) (8, 9) are commonly used. Depending on their design, analgesia is either recommended or compulsory in sepsis models (10). Apart from ethical justifications, anesthesia is critical given that untreated pain hampers metabolic and immunological functions and impairs protective behaviors, such as reduced activity and food intake, thereby altering the disease phenotypes (11–13). The limited availability and immunosuppressive effects of opioids necessitate an exploration of non-opioid alternatives that are in line with animal welfare and ethical guidelines (14). However, the use of non-opioid analgesics (N-OA) in sepsis is controversial due to their potential confounding immunomodulatory properties (15). Thus, it is vital to characterize these immunomodulatory effects of N-OA in relevant sepsis models.

Oral medication is optimal for peri- and post-operative pain control, and nonsteroidal anti-inflammatory drugs (NSAIDs), like Meloxicam, are commonly applied for this purpose. However, by inhibiting the cyclooxygenase (COX)-1/2, Meloxicam also demonstrates a considerable anti-inflammatory action requiring testing in the setting of sepsis (16, 17). The non-opioid Metamizole (Dipyrone), functioning in a similar way to conventional NSAIDs, is a pro-drug from the Ampyrone sulfonate family of medicines and has weak anti-inflammatory and anti-thrombotic properties (18, 19).

Currently, research addressing the use of N-OA analgesics in sepsis models is relatively scarce. This study investigates the impact of two specific N-OA regimens upon several pathophysiological and immune-inflammatory endpoints in the acute phase of a PCI sepsis model. Such a head-to-head characterization of Meloxicam and Metamizole has three-tier benefits: i) identifies strengths/weaknesses of each drug, ii) aids in better decision-making in the preclinical studies, and iii) provides a deeper mechanism-of-action understanding of those drugs.

## 2 Materials and methods

### 2.1 Ethical statement

All experiments were performed in line with German laws and regulations. The animal experiments followed approved protocols (Thuringian State Administrative Office, Thuringia, Germany, Reg. No. UKJ-20-025). The human material sampling was approved by the Ethical Board of the Friedrich-Schiller University Jena (Reg. No. 2019-1413-Material).

### 2.2 Human fecal slurry

We recruited healthy volunteers aged 18 to 50, both male and female, for our study. Exclusion criteria included vegetarian or vegan, chronic immunosuppression or intestinal/autoimmune diseases, recent antibiotic or immunosuppressive drug use, international travel within two months, or indigestion within two weeks. Participants provided stool samples within a 2-hour morning window, which were then weighed and kept on ice for a maximum of 3 hours before processing. We measured Thioglycollate medium (#T9032, Merck, Germany) and added 10% barium sulfate (#11432, Fluka, Germany) based on the total fecal weight. After blending to achieve a homogeneous suspension, we added 10% sterile Glycerol and mixed thoroughly. The material was aliquoted into sterile containers, frozen on dry ice, and stored at -80°C until analysis. Six frozen samples underwent microbial characterization at the clinical microbiology lab of the Jena University Hospital using approved biochemical and LC-MS protocols. Additionally, three samples underwent PCR testing for clinically relevant viruses.

### 2.3 Murine peritoneal contamination and infection sepsis model and survival analysis

#### 2.3.1 Animals and housing

The animals of this study were C57BL/6J male and female mice, aged between 8 and 12 weeks, and housed in individually ventilated cages (IVC) under specific-pathogen-free (SPF) conditions in the Central Experimental Animal Facility of Jena University Hospital, Friedrich-Schiller-University, Jena, Germany. The mice were maintained under 12-h light/dark cycles with a 20-minute dim phase at 22°C and humidity of 55% ± 10%. The mice were given a week to acclimatize in the animal facility before being used in the experiment.

#### 2.3.2 Sepsis model and supportive therapy

All animal protocols reported in the study adhered to the recommended guidelines for preclinical sepsis studies (10). The animals were not randomized, materials were not blinded for the experiment, and were allocated by an independent scientist to the groups. Whenever possible, the assignment to a group was cage-wise, maintaining a near-equal gender distribution. Each experiment was performed in at least three independent runs. The order in which animals received treatment was varied between runs, reducing the risk of systematic biases. The peritoneal contamination and infection (PCI) model was employed to induce polymicrobial sepsis through intraperitoneal injection with human feces suspension prepared as described above (7, 20). The human fecal suspension was mixed with Ringer solution (3 µL Ringer per mg fecal suspension) and vortexed directly before use. A dose range of human fecal suspension (60 µL, 80 µL, 100 µL, and 120 µL) was tested in a survival experiment to calibrate the material. For ethical considerations, Metamizole was administrated to this dose-finding cohort for analgesia. Surviving animals were sacrificed 14 days after infection, terminating the experiment.

The 100 µL dose caused life-threatening sepsis and was chosen for further analgesic experiments. Since there were no significant survival changes after day 7 in the dose calibration experiments, further survival studies were terminated on that day. Since rodent drug metabolism is increased compared to humans, all analgesics were applied according to veterinarian standards, ensuring a therapeutic effect (21). Metamizole (N=45, orally with 2.5 mg per mouse, 4 times per day) (22) or Meloxicam (N=47, orally with 2.5 mg per mouse, 2 times per day) (23) were administered from the beginning of the experiment. A Non-Analgesic group (Non-A) was included as a control (N=46). During the 7-day sepsis period, mice were weighed daily and injected subcutaneously with Meropenem at 25 mg kg^-1^ in Ringer Solution (10 mL kg^-1^) every 12 h, starting 6 h after infection.

#### 2.3.3 Monitoring

Additionally, their rectal temperature (24), Grimace Scale (GS) (25), and Clinical Severity Score (CSS) (**Supplementary Table 1**) (7, 20, 26), were monitored up to six times a day depending on the animals’ condition (**Supplementary Figure 1**). GS, first described by Dale J Langford et al. in 2010 (25), was applied four times daily to evaluate pain. GS discriminates four pain signs (orbital tightening, nose bulge, check bulge, ear position, and whisker change), each scored with 0, 1, or 2 points in case of no, mild, or severe changes. CSS, introduced by Gonnert et al. in 2012, was utilized to measure sepsis severity (26). The score consists of four variables: the animal’s activity, grooming, and clinical signs like diarrhea and posture. Each scored between 1 and 4, with 1 referring to healthy and 4 to severe signs.

#### 2.3.4 Endpoints

The points can be added up to classify sepsis severity, and a point score of 4 equals no signs of illness, >4 mild symptoms, >8 severe symptoms, and >12 to an animal in shock or lethargy where death within 3 hours is imminent. Therefore, the survival experiment sacrificed animals, reaching a score of >12.

### 2.4 Tissue harvest

24-hour post-PCI, a subgroup of septic mice for None-analgesic, Metamizole, and Meloxicam and a sham group with saline intraperitoneal injection (N=12 of each cohort) were euthanized. Within the 24 h infection period, no animals reached an endpoint. Under isoflurane deep anesthesia, the peritoneal lavage fluid (PLF) was obtained by gently massaging the peritoneal cavity for 30 seconds with 2 mL sterile PBS containing 5 mmol L^-1^ EDTA. The blood samples were collected through cardiac puncture using EDTA-coated tubes, and the liver and spleen were subsequently collected. The harvested organs were either freshly processed for single-cell suspension and running flow cytometry or snap-frozen in liquid nitrogen and stored at −80°C for further analysis.

### 2.5 Bacterial burden

For bacterial burden analysis, samples of bioliquids (blood and PLF) and solid tissues (liver and spleen) were snap-frozen and stored at -80°C. Bacterial DNA was isolated with the ZymoBIOMICS DNA Microprep kit (#D4301, ZYMO research, USA), and bacterial burden quantification was performed using the Femto Bacterial DNA quantification kit (#E2006, ZYMO research, USA) via quantitative polymerase chain reaction (qPCR) with Rotor-gene Silber (QIAGEN, Netherlands). Based on the standard curve, the final concentration of bacteria was normalized per mL for biofluids or mg for solid tissues. Colony-forming units (CFUs) were determined in PLF by serially diluting in sterile DPBS and plating on plate-counting Agar (APHA, ISO 4833:2003, for microbiology, Carl Roth, Germany). All samples were prepared in triplicates, and CFUs were counted with Schuett Colony Quant colony counter after incubating plates at 37°C for 48 h.

### 2.6 Immune cell-type and CXCR2 expression across tissues

Single-cell suspensions were prepared freshly from four different tissues post-harvesting on ice. The blood was centrifuged at 1,000 rcf for 10 minutes to obtain the plasma supernatant. Leukocytes in blood were isolated using gelatin sedimentation (#b57, Gelafundin 40 mg mL^-1^, Braun Medical, Switzerland) for 30 minutes at room temperature, followed by derived supernatant centrifugation at 500 rcf at 4°C for 5 minutes to obtain immune cell pellets. Fresh PLF immune cells were centrifuged at 500 rcf for 10 minutes. The liver tissue was homogenized in a single cell dissociator (DSC-400, RWD, China) and filtered using a 70-µm cell strainer (#431751, Corning, USA) into a 50 mL centrifuge tube containing 10 mL ice-cold PBS with 5 mmol L^-1^ EDTA. The derived liver cell suspension was then centrifuged at 40 rcf for 4 minutes at 4°C to separate the majority of hepatocytes, while the supernatant was collected into a new falcon tube and centrifuged at 1,000 rcf for 5 min to remaining non-parenchymal cells. These cells were resuspended in 33% Percoll (GE17-0891-01, Sigma-Aldrich, Germany) and spun at 800 rcf for 30 minutes at room temperature to obtain the non-parenchymal cell pellets (27). The spleen was cut into pieces and strained with a plunger through a 70-µm cell strainer into a 50 mL conical tube. The splenocytes were then pelleted via centrifugation at 500 rcf for 5 minutes.

Immune cells from different organs were separately collected as described and washed with cold Staining buffer (#554656, BD Bioscience, USA) at 500 rcf for 5 minutes at 4°C. All subsequent procedures were conducted under 4°C. Cells were counted and resuspended in 50 uL staining buffer with 10^6^ cells, incubated with Fc receptor blockade (#130-092-575, Miltenyi Biotec, Germany) for 10 minutes, followed by incubation with primary surface antibody cocktails, including PerCP-CD11b (#101229, BioLegend, USA), APC-Ly6G (#127613, BioLegend, USA), BV421-F4/80 (#123131, BioLegend, USA) or BV421-Ly6C for the blood (#128031, BioLegend, USA), FITC-CXCR2 (#149309, BioLegend, USA), for 30 minutes in the dark. The immune cells were washed with Staining buffer and pelleted by centrifugation at 500 rcf for 5 minutes. Cells were then fixed and permeabilized simultaneously with Cytofix/Cytoperm solution (#1184665, BD Bioscience, USA) for 20 minutes and then washed in Perm/Wash buffer (#1291074, BD Bioscience, USA). The cells were pelleted and resuspended in 50 uL of Perm/Wash buffer containing anti-cytokine intracellular antibody cocktail, including BV605-IL-10 (#505031, BioLegend, USA), BV785-IFN-γ (#505837, BioLegend, USA), BV510-TNF-α (#506339, BioLegend, USA), PE-IL-1α (#130-120-955, Miltenyi Biotec, Germany), and incubated for 30 minutes in the dark. The cells were washed and resuspended with 1x Perm/Wash buffer, then analyzed by a flow cytometer CytoFlex (Beckman, USA). The cytometric data was analyzed with FlowJo 10.8.1 (BD Bioscience, USA). Initially, FSC and SSC were applied to identify alive singlets. Subsequently, myeloid cells were recognized as CD11b^+^, Neutrophils were characterized as CD11b^+^ Ly6G^+^ F4/80^-^, while CD11b^+^ Ly6G^-^ F4/80^+/low^ were considered as macrophages, CD11b^+^ Ly6G^-^ Ly6C^+^ were considered as blood monocytes, other myeloid cells were identified as CD11b^+^ Ly6G^-^ F4/80^-^, those cell types were displayed with positive percent of alive singlet cells, with the mean fluorescence intensity for internal cytokines (**Supplementary Figure 2**).

### 2.7 Quantification of inflammatory cytokines and chemokines across tissues

The plasma and cell-free supernatant of PLF was frozen at -80°C. The liver and spleen were weighted and mechanically dissociated with lysis buffer with 10% NP-40 (I8896, Sigma-Aldrich, USA) plus 0.05M EDTA (Nr.8040.1, Carl Roth, Germany) in 1x Tris-buffered saline (#2992224, Merck Millipore, Germany) containing protease inhibitor (#481759, Roche, Switzerland) using TissueLyser at 25 Hz for 3 minutes at room temperature. The resulting mixture was centrifuged at 10,000 rcf for 10 min at 4°C to get supernatant of cell-free lysates of liver and spleen. The inflammatory cytokines and chemokines in the cell-free supernatant of various tissues were quantified using the LEGENDplex Mouse inflammation panel (#740150, BioLegend, USA), following the manufacturer’s instructions. This panel allows simultaneous quantification of 13 mouse cytokines, including IL-1α, IL-1β, IL-6, IL-10, IL-12p70, IL-17A, IL-23, IL-27, monocyte chemoattractant protein-1 (MCP-1), interferon-beta (IFN-β), interferon-gamma (IFN-γ), and granulocyte-macrophage colony-stimulating factor (GM-CSF). Bead-assisted multiplex cytokine profiling was evaluated using CytoFlex (Beckman, USA), and the data were analyzed using LEGENDplex software.

### 2.8 mCXCL2 and PGE2 ELISA

We measured levels of macrophage inflammatory protein-2 (MIP-2, also known as CXCL2, #426062723, Invitrogen, USA) and prostaglandin E2 (PGE2, #KGE004B, R&D Systems, USA) in bioliquids, including plasma/circulating and PLF, using a quantitative ELISA assay. Optical densities were recorded at specific wavelengths using an ELISA-dedicated instrument called EnSpire multimode plate reader (Perkin Elmer, USA) according to the absorbance wavelength (450 nm for CXCL2, 450 nm corrected with 540 nm for PGE2). Concentrations of cytokines were determined using the standard curve with a four-parameter algorithm. Each sample was quantified twice, and the average value of both replicates was calculated.

### 2.9 IL-6 stratification protocol

In this study, we observed a significant elevation in plasma/circulating IL-6 levels at 24h post-PCI across all sepsis groups compared to the sham controls, regardless of analgesic treatment. Consequently, we identified the median circulating IL-6 level in Non-A septic mice (15,052 pg µL ^-1^) as the cutoff. The circulating IL-6 levels above or below the designated cutoff in all septic mice were then recognized as a predictor of death and survival, termed as the high IL-6 responders (Non-A septic mice: n=6, Metamizole treated septic mice: n=7, Meloxicam treated septic mice: n=5) and low IL-6 responders (Non-A septic mice: n=6, Metamizole treated septic mice: n=5, Meloxicam treated septic mice: n=7) (28–30).

### 2.10 Data analysis and statistics

#### 2.10.1 Software

The statistical analysis utilized RStudio (Version 2022.12.0 + 353). The following R packages were utilized to process and visualize the data: nphRCT, survival, survminer, forestmodel, ggpubr, ggplot2, pheatmap.

#### 2.10.2 Statistical hypothesis and power analysis

The statistics were applied based on the null hypothesis that each pair of group means is equal, i.e. there is no difference between animals that did not receive analgesia or were treated with either Meloxicam or Metamizole. The alternative hypothesis was that at least one pair of group means is unequal. In all figures, three groups were compared: sepsis with non-analgesic, sepsis with Metamizole, sepsis with Meloxicam.

A power analysis based on estimates derived from previous experiments was performed. Experiments were performed in several independent runs, with interim evaluations performed after each run to ensure the ethical use of animals.

The power analysis for the survival experiments determined the necessary sample size for differentiating between moderate sepsis (with a 20% 48-hour mortality rate) and severe sepsis (with a 50% 48-hour mortality rate). The objective is to detect a 30% difference in 48-hour mortality with a statistical power of at least 0.8 and a significance level of 0.05 (type I error). The power analysis was based on a two-sided log-rank test. Key parameters included a mortality ratio of 2.50 (treatment group mortality rate divided by control group mortality rate), with the control group having a 20% mortality rate and the treatment group having a 50% mortality rate. The study assumed an accrual time and total observation time of 2-time units each, with no loss of subjects or group switching considered. Under these conditions, a power of 0.8037 was achieved, slightly exceeding the minimum required power of 0.8. The analysis concluded that 47 animals per group are needed.

To investigate differences in the immune response 24 h after infection, MCP-1 levels, a cytokine elevated in the PCI model after immunosuppression, served as a surrogate marker across the experimental groups (31). The analysis was based on a non-parametric comparison between all groups, with a target of detecting a minimum 10% difference between any two groups considered physiologically relevant. The power analysis aimed for a type I error rate of 0.05 and a statistical power of at least 0.8. The analysis resulted in a required sample size of 12 animals per group. Using the Kruskal-Wallis test, the power analysis simulation confirmed that this sample size would achieve an any-pairs power of 1.000 and an all-pairs power of 0.809, meeting the predetermined statistical criteria.

#### 2.10.3 Statistics

Kaplan-Meier survival curves assessed survival data, with P-values calculated using the log-rank test. Non-proportional hazards crossing Non-A and metamizole-treated septic mice were tested with a modestly weighted log-rank test (mWLRT), and Cox proportional hazards were applied for the forest model. A normality test was conducted using the Shapiro-Wilk test (α=0.05), ANOVA with Tukey’s HSD for parametric data, or Kruskal-Wallis test with Wilcoxon Mann-Whitney U tests and Holm-Bonferroni correction (HB) for non-parametric analysis, was applied afterward for a multiple-comparison test. The group means and standard deviation (SDs) or medians and interquartile ranges (IQRs) were calculated for each parameter of interest and time point. A P-value below 0.05 indicated a significant difference (* P<0.05, ** P<0.01, *** P<0.001, **** P<0.0001).

## 3 Results

### 3.1 Calibration of the human stool suspension for PCI sepsis model

**Figure 1:**
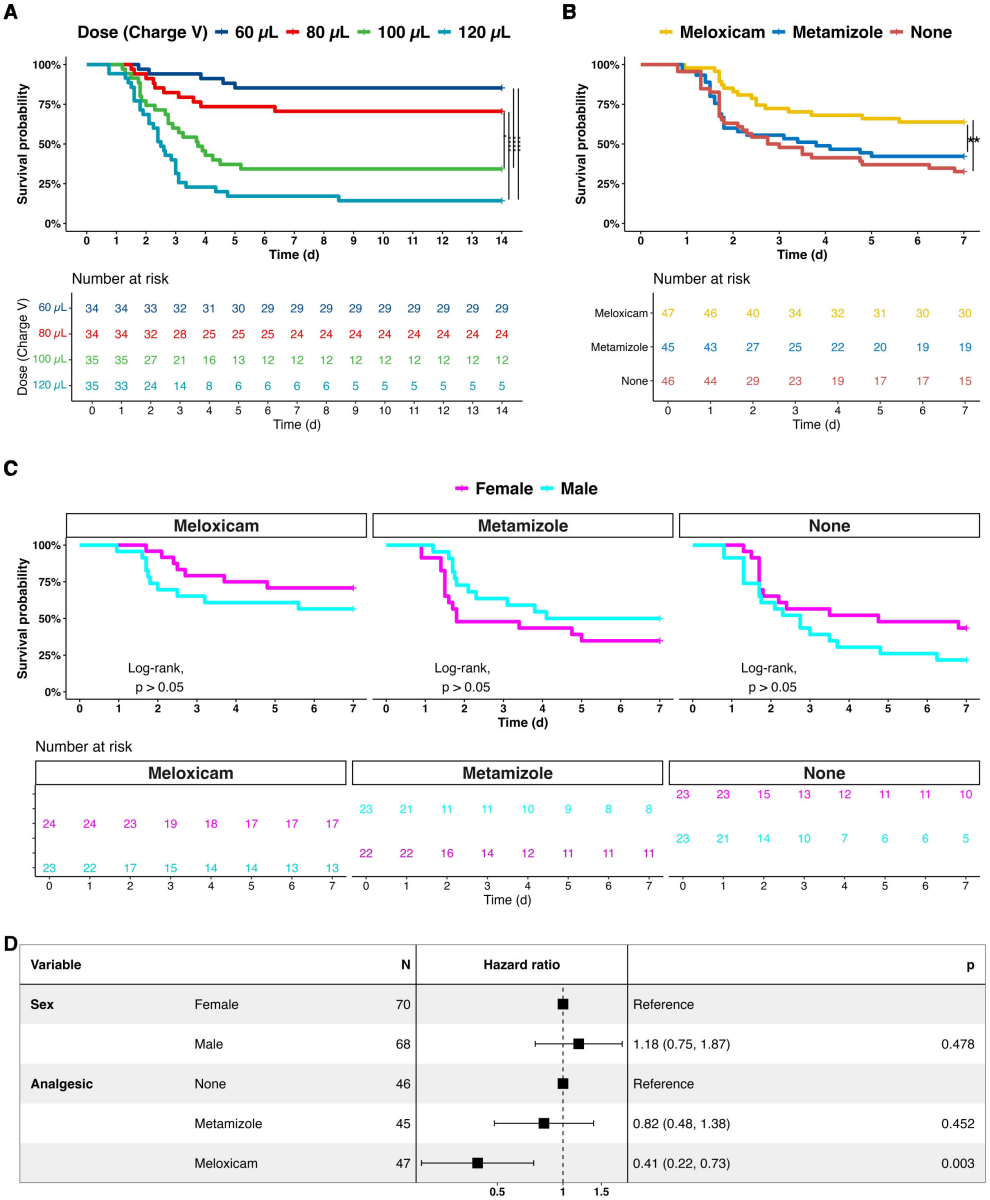
Survival analysis of septic mice treated with adjuvant non-opioid analgesics. **(A)** Human feces dose-effects on the sepsis progression and 14-day survival. **(B)** Comparative survival analysis of analgesics-treated septic mice of Meloxicam, Metamizole, and without adjuvant analgesia (None) 7 days after infection. **(C)** Comparative survival analysis of female and male septic mice with analgesia. **(D)** A forest plot of sex and analgesics for septic mice. A log-rank test with pairwise comparison and Cox’s proportional hazard model was employed for statistical testing, * P < 0.05, ** P < 0.01, *** P < 0.001, **** P < 0.0001.

The infectious material for the PCI model (human feces) consisted of 2*10^12^ bacteria per mL. The two dominant bacteria populations identified by culture and biochemical characterization were *Enterococcus faecilis* (40%) and *Escherichia coli* (35%) (**Supplementary Figure 1**) (7). In a 14-day survival experiment, four doses of human feces ranging from 60, 80, 100, and 120 µL were administered to evaluate the progression of infection and to establish appropriate dosages corresponding to distinct severity levels. For ethical considerations, Metamizole was used for pain release in this cohort. Throughout the 14-day post-PCI period, mortality rates of septic mice were observed to be comparatively lower at 60 µL and 80 µL doses than at 100 µL and 120 µL doses, respectively. Notably, there was no significant survival distinction between the 60 µL and 80 µL doses nor between the 100 µL and 120 µL doses. The peak mortality occurred between days 1 and 3, plateauing after 7 days (**Figure 1A**). Sex had no significant impact on survival. Based on the model’s kinetic, day 7 was defined as the end of observation, and 100 µL human fecal suspension was administered to produce a robust and early acute-phase response for subsequent analgesic experiments.

### 3.2 Meloxicam reduced the mortality of septic mice

We were first interested in whether NSAIDs would have a measurable effect on the survival of PCI mice. Therefore, mice were categorized into three groups: those receiving no analgesic treatment (Non-A), those treated with Meloxicam, and those treated with Metamizole. The data revealed that the 7-day mortality rate was 67% in the group without analgesics. Compared to the Non-A group, survival decreased to 58% (P=0.495) in the Metamizole-treated group and to 36% (P=0.0059) in the Meloxicam-treated group (**Figure 1B**).

Further analysis was conducted to determine the Hazard Ratios (HR) for Meloxicam and Metamizole compared to the Non-A group. HR for Meloxicam was 0.41 (95% CI: 0.22 - 0.73, P = 0.003), indicating a substantial reduction in mortality risk compared to the Non-A group. In contrast, HR for Metamizole was 0.82 (95% CI: 0.48 - 1.38, P = 0.452) (**Figure 1C**). Additionally, sex had no effect on survival, with an HR of 1.18 (95% CI: 0.75 - 1.87, P = 0.478) (**Figure 1D**).

### 3.3 Analgesics attenuated pain but did not modulate clinical severity in septic mice

**Figure 2:**
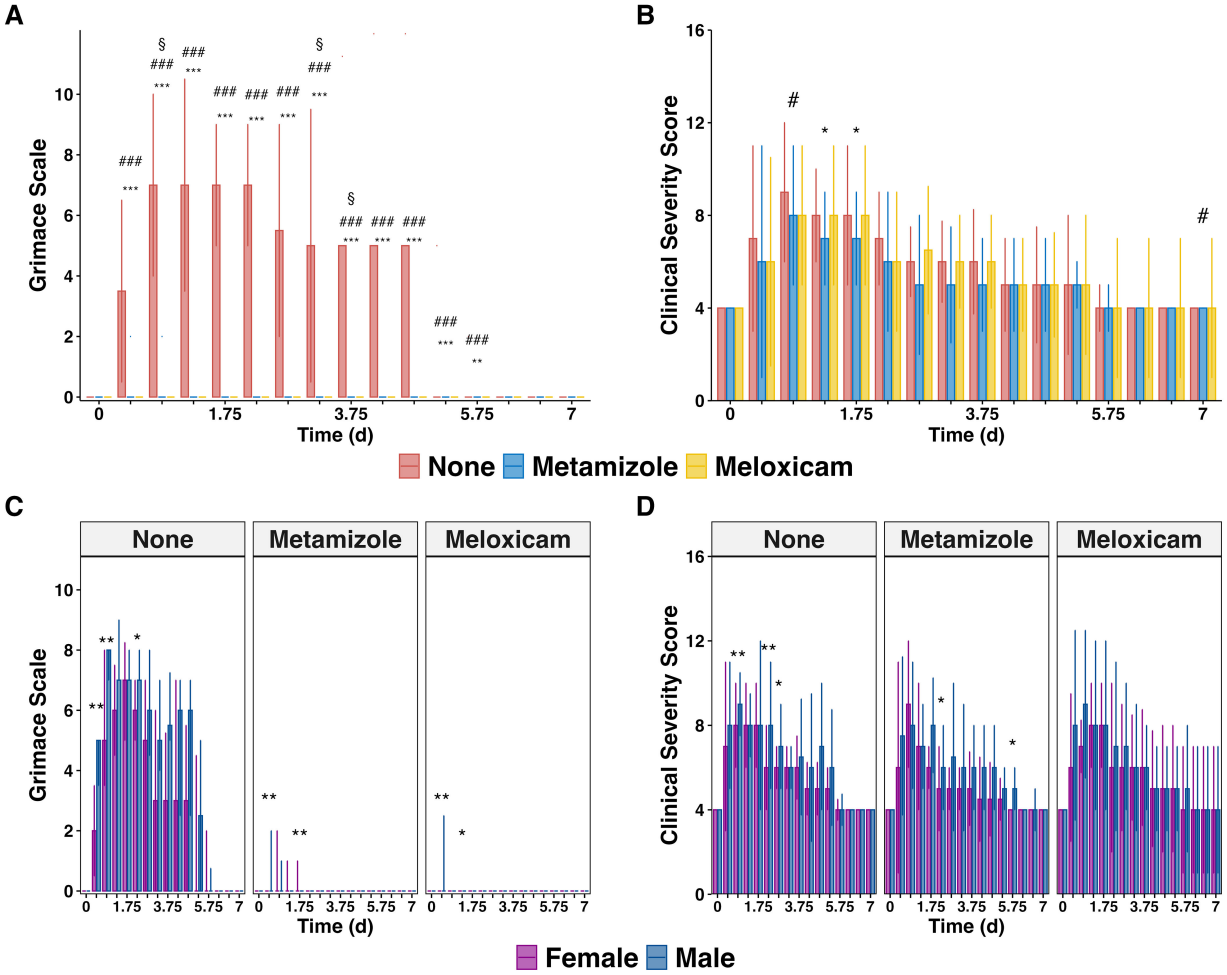
Effectiveness of pain relief and clinical severity scores in mice suffering sepsis. **(A)** Median cumulative Grimace Scale (GS) plus IQR of analgesics-treated septic mice and **(B)** Median cumulative Clinical Severity Score (CSS) plus IQR of analgesics-treated septic mice. **(C)** GS and **(D)** CSS are grouped by gender. The Wilcoxon test with HB correction was employed for statistical testing, * P < 0.05, ** P < 0.01, *** P < 0.001, the same for # and §. Specific significant symbols for **(A, B)**: * Sepsis with no analgesics versus Sepsis with Metamizole, # sepsis with no analgesics versus Sepsis with Meloxicam, § Sepsis with Metamizole versus sepsis with Meloxicam.

The grimace scale (GS) results revealed a high pain level in Non-A septic mice, especially in the early PCI phase. Both NSAIDs, Meloxicam and Metamizole, effectively alleviated GS levels in this PCI sepsis model (**Figure 2A**). Interestingly, the administration of analgesics only marginally affected the Clinical Severity Score (CSS), when compared to Non-A septic mice. Only on days 1.25 (adj.P=0.024) and 1.75 (adj.P=0.022) in the Metamizole group and on days 0.75 (adj.P=0.038) and 7 (adj.P=0.046) in the Meloxicam group CSS levels significantly decreased (**Figure 2B**). Notably, male Non-A septic mice displayed higher pain and CSS than their female counterparts evidenced by higher GS on days 0.25 (P=0.0026), 0.75 (P=0.008), and 2.25 (P=0.0337) as well as higher CSS on days 0.75 (P=0.0039), 2.25 (P=0.0094), and 2.75 (P=0.0279). However, both Metamizole and Meloxicam treatments did not exhibit any sex preference regarding pain and illness severity (**Figures 2C, D**).

### 3.4 Meloxicam did not modulate sepsis-induced weight and temperature loss

**Figure 3:**
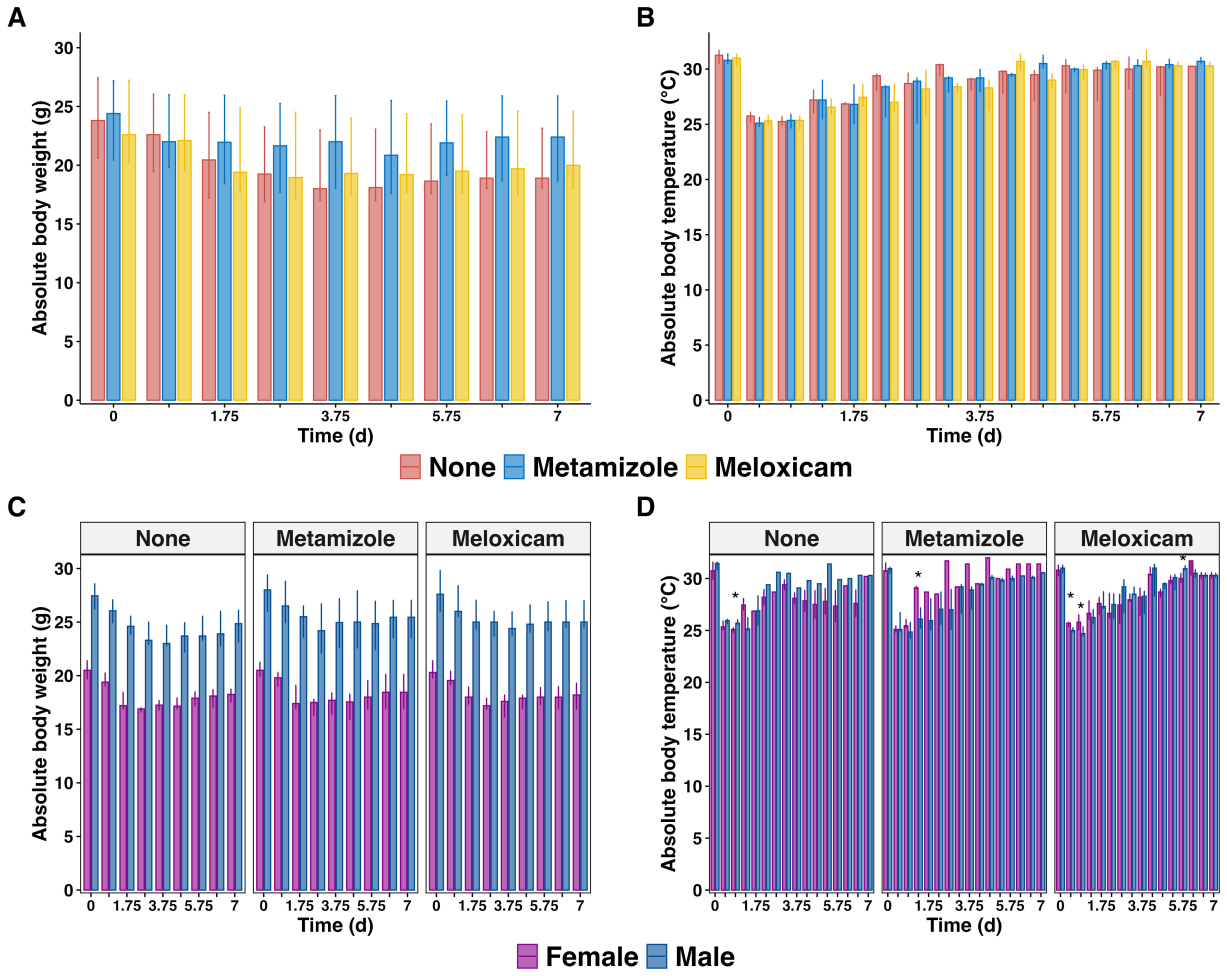
Body weight and temperature changes in septic mice treated with analgesics. **(A)** Median of absolute body weight changes plus IQR of analgesicstreated septic mice and **(B)** grouped by sex **(C)** The median body temperature plus IQR of analgesics-treated septic mice and **(D)** were grouped by sex. The wilcoxon test with HB correction was employed for non-parametric data, * P < 0.05.

The progression of sepsis in mice is characterized by significant changes in body weight and temperature. The body weight of septic mice continued declining until day 5, with a gradual recovery observed afterward. However, the mice failed to fully regain their initial body weight until 7 days post-sepsis. NSAIDs treatment did not affect the body weight changes in septic mice (**Figure 3A**), including unaltered circadian rhythm variations in body temperature. The body temperature of septic mice rapidly decreased reaching nadir on day 1 post-PCI, and recovered to its initial level by day 7 post-sepsis, irrespective of analgesic treatment (**Figure 3B**). Notably, male mice exhibited a more severe illness during the acute PCI phase showed only transient differences in their body temperature loss compared to females: on day 0.75 (P=0.026) in the Non-A sepsis group, day 1.25 (P=0.017) on Metamizole treatment, day 0.25 (P=0.037), 0.75 (P=0.019), and 5.75 (P=0.049) on Meloxicam treatment (**Figures 3C, D**).

### 3.5 Meloxicam increased the bacterial burden in the blood and peritoneum

**Figure 4:**
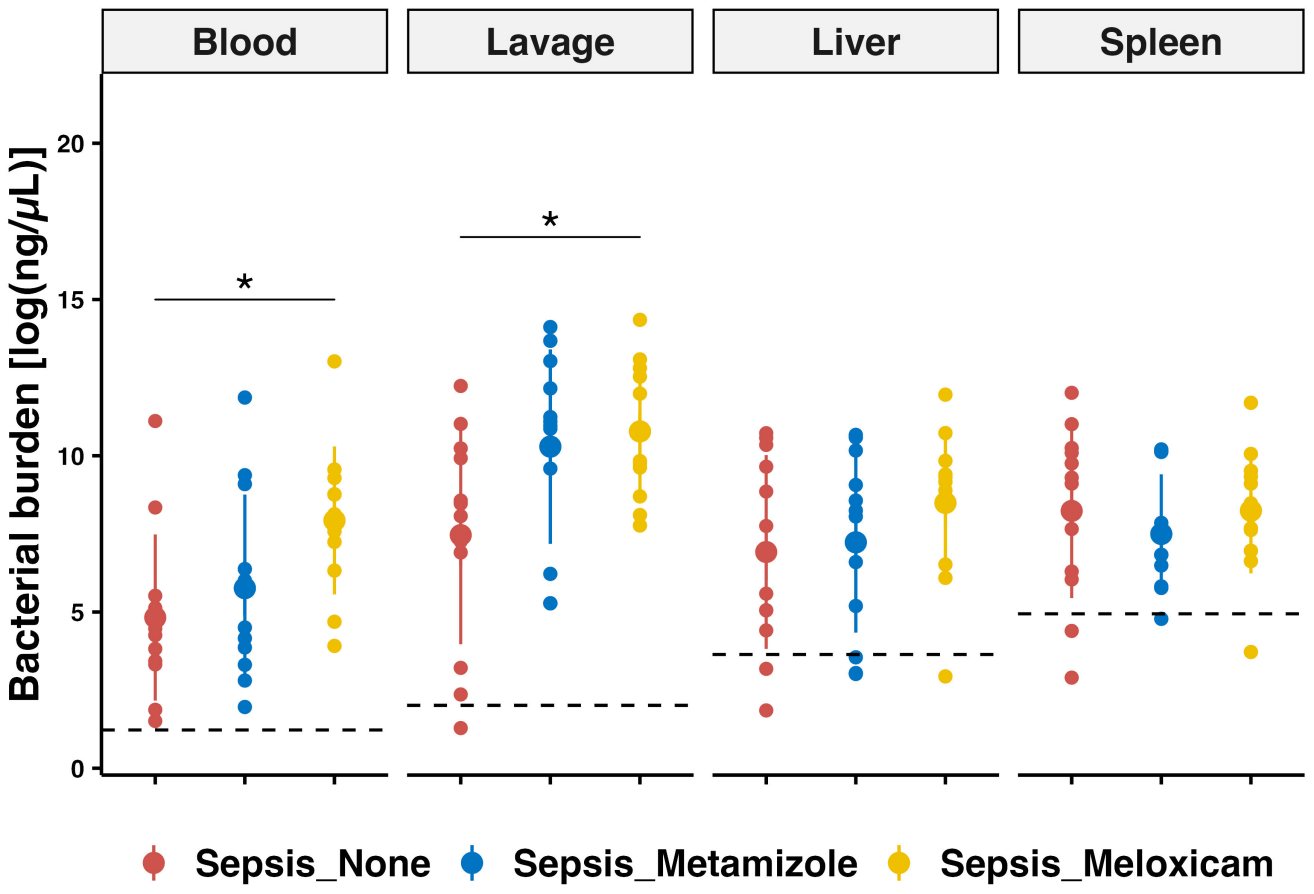
PCR-based bacterial quantification in tissues of septic mice with analgesic regimes. Bacterial quantification was performed using quantitative PCR for blood, peritoneal lavage, liver, and spleen. The mean for mice from sham group is represented by a black dashed line. Errorbar for mean plus SD and dots showing individual animals. Each n=12 for the Sham mice, Non-A septic mice, Metamizole treated septic mice, and Meloxicam treated septic mice. ANOVA with TukeyHSD correction was employed for those parametric data, * P < 0.05.

Clinical evidence did not reveal major differences with analgesic treatment that could be directly attributed to alterations in survival. Thus, we next examined whether Metamziole or Meloxicam impacted bacterial clearance. The differences between Meloxicam and Metamizole were less pronounced, while there was a robust tendency towards a higher pathogen load in the Meloxicam-treated groups. Notably, bacterial counts of blood and peritoneum were only significantly increased in the Meloxicam-treated sepsis group compared to the Non-A ones (3.23-fold, P=0.0208, 95% CI: 0.41 – 5.80, and 2.77-fold, P=0.0264, 95% CI: 0.34 – 6.30) (**Figure 4**, **Supplementary Figure 3**).

### 3.6 Meloxicam modified the tissue immune phenotype in sepsis

The circulating IL-6 stratification method provided insights into distinct inflammation mediator patterns in high and low IL-6 response groups across four tissues. In both the circulating and peritoneal lavage of septic mice, we detected elevated levels of various cytokines compared to the sham mice, which included IFN-γ, IL-10, IL-1α, IL-6, MCP-1, MIP-2, and TNF-α. Additionally, the peritoneal lavage of all sepsis groups exhibited increased PGE2, IL-27, IL-1β, and IL-12p70 levels.

**Figure 5:**
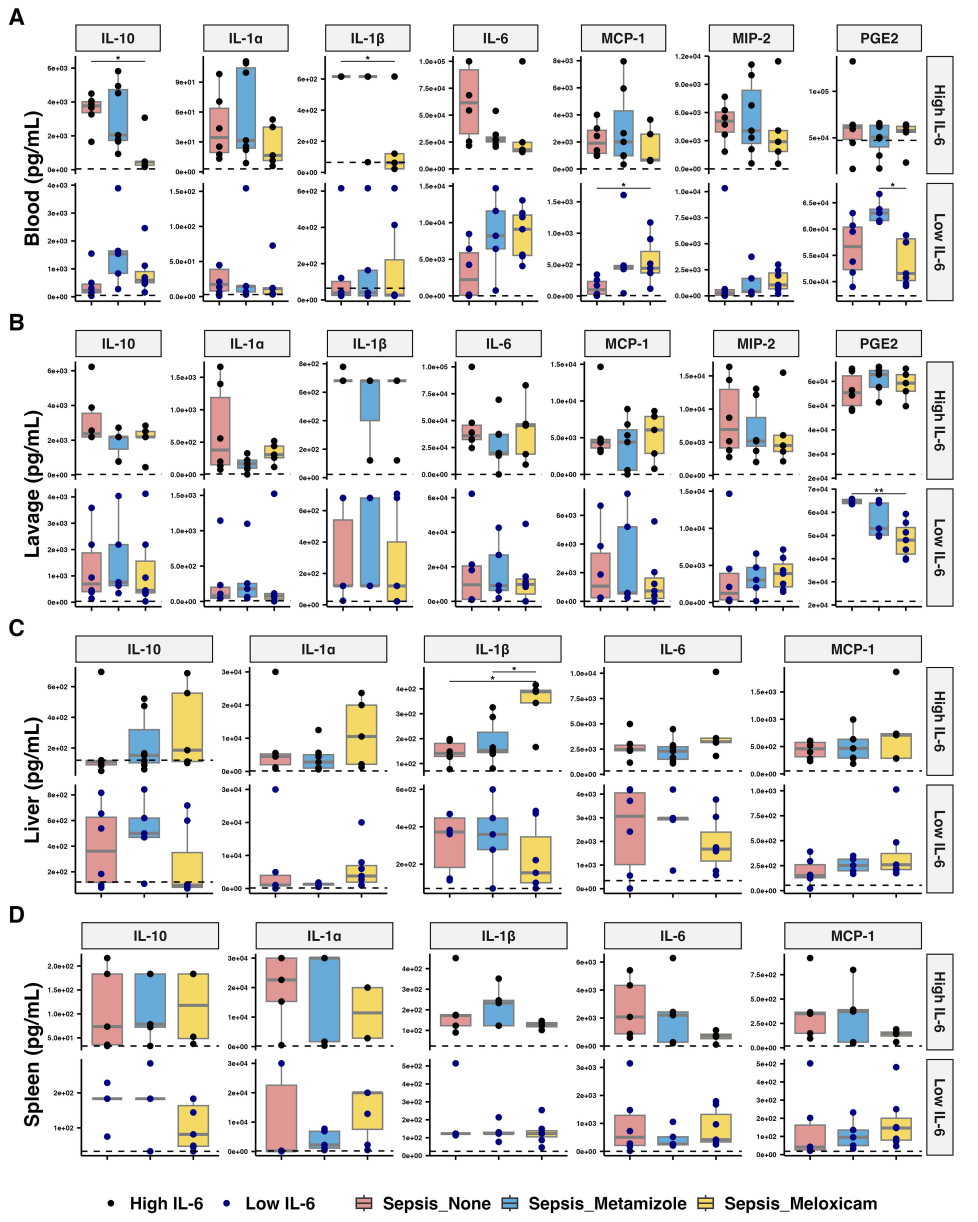
Inflammatory mediators’ levels across tissues of septic mice with analgesic. The concentration of inflammatory cytokines in **(A)** plasma, **(B)** peritoneal lavage fluid, **(C)** liver, and **(D)** spleen of analgesics-treated septic mice. The median for mice from sham group is represented by a black dashline. Sham mice: n=12; Low IL-6 response (Non-A septic mice: n=6, Metamizole treated septic mice: n=5, Meloxicam treated septic mice: n=7); High IL-6 response (Non-A septic mice: n=6, Metamizole treated septic mice: n=7, Meloxicam treated septic mice: n=5). Boxes depict the median and IQR, with dots for individual animals. Wilcoxon test with HB correction was employed for P-value calculation, * P < 0.05.

Meloxicam treatment lowered circulating IL-10 (7.46-fold, adj.P=0.026) and IL-1β (9.96-fold, adj.P=0.044) levels in high IL-6 responders compared to the Non-A group, while circulating MCP-1 (4.37-fold, adj.P=0.024) increased with the Meloxicam-treated in low IL-6 responders. Circulating IL-23 (31.38-fold compared to Non-A group, adj.P=0.03) and IL-27 (13.79-fold, adj.P=0.035) levels rose in high IL-6 responders from the Non-A septic groups and with Metamizole treatment but not in Meloxicam groups. Meloxicam treatment in low IL-6 responders septic mice decreased PLF PGE2 (119.22-fold, adj.P=0.01) levels compared to Non-A sepsis groups, while circulating PGE2 increased in the Metamizole treated low IL-6 responders septic group compared to Meloxicam-treated mice (1.11-fold, adj.P=0.017) (**Figures 5A, B**, **Supplementary Figure 4**).

Meloxicam-treated sepsis mice in high IL-6 responders had increased liver IL-1β levels compared to Metamizole-treated ones (2.25-fold, adj.P=0.030). Minimal changes were observed in other cytokines, including GM-CSF and IFN-β, across all groups (**Figures 5C, D**, **Supplementary Figure 4**).

### 3.7 Tissue immune infiltration in sepsis was not inhibited by analgesic treatment

**Figure 6:**
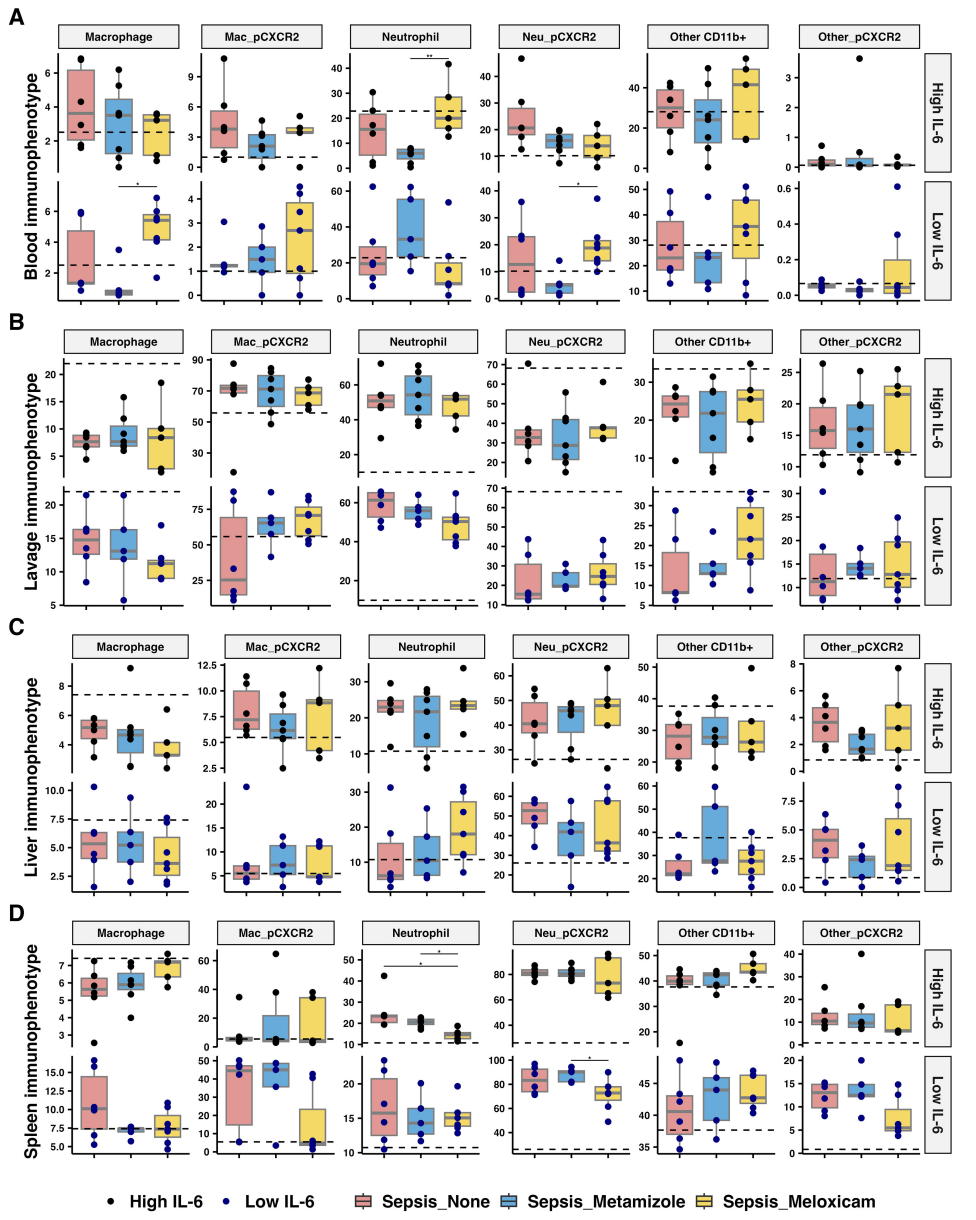
Immunophenotype across tissues of septic mice treated with analgesics. Percentage of myeloid cells, including macrophage, neutrophils, and other CD11b+ myeloid cells, and CXCR2 expressed in respective cells from **(A)** blood via heart puncture, **(B)** peritoneal lavage, **(C)** liver, and **(D)** spleen of analgesics-treated septic mice. The median for mice from sham group is represented by a black dashline. Sham mice: n=12; Low IL-6 response (Non-A septic mice: n=6, Metamizole treated septic mice: n=5, Meloxicam treated septic mice: n=7); High IL-6 response (Non-A septic mice: n=6, Metamizole treated septic mice: n=7, Meloxicam treated septic mice: n=5). Boxes depict the median and IQR, with dots for individual animals. Wilcoxon test with HB correction was employed for P-value calculation, * P < 0.05, ** P < 0.01.

Blood neutrophils increased (3.33-fold, adj.P=0.008) in the high IL-6 responders treated with Meloxicam versus Metamizole. Whereas, in the low IL-6 responders, Meloxicam treatment increased blood monocyte maturation (7.14-fold, adj.P=0.015) and resulted in higher neutrophil CXCR2 expression (2.43-fold, adj.P=0.03) (**Figures 6A, B**). Analgesic treatment did not inhibit cellular infiltration of the peritoneum at 24 hours after infection. Meloxicam treatment (vs Metamizole) reduced the spleen neutrophils (1.42-fold, adj.P=0.024) in the high IL-6 responders but reduced CXCR2 surface expression for low IL-6 responders (1.25-fold, adj.P=0.03) (**Figures 6C, D**). Analgesic treatment did not alter the internal cytokine levels, including IFN-γ, IL-10, IL-1β, and TNF-α, at 24 hours after infection (**Supplementary Figure 4**).

## 4 Discussion

### 4.1 Analgesic effects and pathophysiology changes on sepsis

In this study, we compared the effects of Meloxicam and Metamizole in a clinically relevant mouse model of polymicrobial sepsis. Our findings suggest that Metamizole, compared to Meloxicam, constitutes a superior analgesic regimen in the PCI model, given its minimal effects on the survival and immune system.

Meloxicam decreased the mortality of septic mice compared to the Non-Analgesic or Metamizole-treated mice. Such survival benefits of NSAIDs have been shown 2% - 12% decrease in patients with severe sepsis with low-dose Aspirin (100 mg per day) treated and pre-treated (32–34), but not for patients older than 70-year (35), which was achieved through the anti-inflammatory lipoxin and NF-κB pathways due to its non-specific COX inhibitory activity and antiplatelet properties (36, 37). The pharmacokinetic and metabolic differences between humans and mice for non-opioid drugs, combined with the variability in dosage, biodistribution, and effects, influence the pain management outcomes of Meloxicam. Meloxicam has a maximum dosage of 15 mg per day for humans and mice (38), while Metamizole is 4000 mg per day for humans and 1000 mg/kg for mice (22, 39). High doses of Metamizole can cause adverse effects in humans, and mice exhibit more excellent resistance to high doses of Metamizole, together with dose-dependent analgesic effects (40, 41).

Metamizole displayed an efficient analgesic but did not impact survival, unlike Meloxicam. In addition to its conventional inhibition of cyclooxygenase (COX) and decreased PGE2 synthesis (42), Metamizole’s potent analgesia is likely associated with central cannabinoid receptor 1 and peripheral neuronal KATP channel opening (43).

Meloxicam markedly increased bacterial burden in the peritoneum and blood of septic mice compared to the Non-Analgesic group. Both analgesic treatments effectively alleviated pain. However, neither significantly altered sepsis’s clinical severity and body weight and temperature loss. This suggests that those parameters were primarily influenced by sepsis rather than the analgesic treatment, indicating a dissociation between pain relief and pathophysiological status.

### 4.2 Immune response and sepsis markers

Sepsis invokes a systemic immune and inflammatory response in the host, including overwhelming inflammatory cytokines, mediators, and immune cell infiltration into tissues. We chose several parameters distinctly associated with sepsis’s pathophysiology, diagnosis, and prognosis. An elevated concentration of circulating IL-6 is an established indicator of an activated inflammatory process and a predictor of unfavorable symptoms and deaths in animal and human sepsis (29, 44).

Following previous examples (30, 45), our study used retrospective IL-6 stratification to better characterize the effects of the tested NO-A in the PCI model. High levels of circulating IL-6 during the acute phase of sepsis are associated with a high risk of mortality, and analgesic treatment could be effective for high responders with excessive immune responses, explaining the reduced mortality observed with meloxicam treatment in PCI sepsis mice.

PGE2 is also a critical lipid mediator in sepsis and could be disrupted by NSAID treatment via COX inhibition (46). However, in low IL-6 responders, meloxicam treatment may have resulted in low levels of PGE2 due to its inhibition of COX-2 (47). One study found that Meloxicam administration in rabbits with antigen-induced arthritis increased neutrophil recruitment and elevated IL-8 in the synovial fluid, with no changes in PGE2 and MCP-1. Additionally, analgesic treatment did not alter immune cell infiltration into tissues in the first 24 hours after infection. This is consistent with findings in COVID-19 patients, in which NSAID treatment suppressed neutralizing cytokines and immune responses such as IL-6, MIP-1, and GM-CSF without affecting the infiltration of innate and adaptive immune cells into the lung (48).

### 4.3 Analgesic considerations and limitations

Despite the advantages of Metamizole over Meloxicam in terms of its minimal modulation of the immune system, Metamizole’s use is limited in some countries due to the rare but severe risk of agranulocytosis and anaphylaxis as side effects in patients (49). However, careful monitoring can mitigate these risks, and no adverse events related to Metamizole have ever been reported in veterinary medicine (50, 51). This study demonstrates a significant strength in its novel investigation of the comprehensive immune response across multiple tissues in a PCI sepsis mouse model treated with analgesics. Our study covers multitier elements of sepsis pathophysiology by focusing on the differential impacts of analgesics on survival, bacterial counts, and immune response. These findings support the use of analgesics in the PCI model, especially for mechanistic studies, with Metamizole emerging as a preferable option over Meloxicam due to its robust pain-relieving but minimal influence on survival and immune response.

A standardized processed batch of frozen stool sourced from healthy non-vegetarian human donors instead of a murine one was employed in this study. Intraperitoneal injection of a defined volume of cecal slurry was administered to the experimental animals, facilitating the establishment of a PCI sepsis model characterized by ease of implementation, high reproducibility, and low variability (7, 20, 31). Our proposed PCI model exhibited comparable hemodynamic and pathophysiological alterations to those observed in human sepsis, even when utilizing cecal slurry obtained from different species, such as mice and rats (52–54). Although humans and mice share approximately 90% of their gut microbiota (55, 56), the proportion of bacteria populations may raise challenges in creating a fully humanized gnotobiotic mouse model, particularly if these bacteria exert species-specific physiological effects. Therefore, further characterization of blood cultures derived from human and rodent stools is needed to better understand polymicrobial-derived sepsis. Additionally, the pathogen community specially obtained from septic patients could possibly be integrated into future investigations to elucidate the specific microbial contributors to translational human sepsis (57).

However, several limitations should be acknowledged. First, the high mortality after 48 hours limited our ability to track time-series changes in immune response across tissues, particularly within the initial 24 hours of high immune and inflammatory responses. Second, we only focused on 2 specific NO-As in a single mouse PCI sepsis model, which restricts us from directly extrapolating our findings to other clinically relevant models and species. Lastly, the absence of quantification of drug concentrations in the blood samples precluded an assessment of the temporal distribution patterns of the two NSAIDs. Addressing these limitations in future research could enhance our understanding of analgesic efficacy and immune modulation in septic conditions.

## 5 Conclusion

Metamizole emerged as the preferred analgesic option for the PCI mouse sepsis model compared to Meloxicam. Metamizole exhibited minimal disruption of immune response dynamics, as evidenced by its limited impact on circulating/tissue cytokines, immune cell infiltration, and bacterial clearance while providing sufficient antinociceptive effects. In contrast, Meloxicam exhibited pronounced immunomodulatory effects, increasing bacterial burden and altering cytokine profiles, particularly in high IL-6 responders. Testing of alternative analgesic regimens across other sepsis models should be addressed in future research to cover the existing knowledge gaps and expand our understanding of the humane design of critical care disease models.

## Data Availability

The raw data supporting the conclusions of this article will be made available by the authors, without undue reservation.
