# Supplementary material for: Metamizole outperforms meloxicam in sepsis: insights on analgesics, survival and immunomodulation in the peritoneal contamination and infection sepsis model
Source: Front Immunol. 2024 Aug 30;15:1432307. doi: 10.3389/fimmu.2024.1432307 (PMC11392727; doi:10.3389/fimmu.2024.1432307)
Supplement: Supplementary file 1 [file DataSheet1.pdf]

Supplementary Material

1 Supplementary Figures and Tables

1.1 Supplementary Figure 1. Analgesic treatment on Peritoneal Injection and Contamination (PCI) sepsis model on C57BL/6J

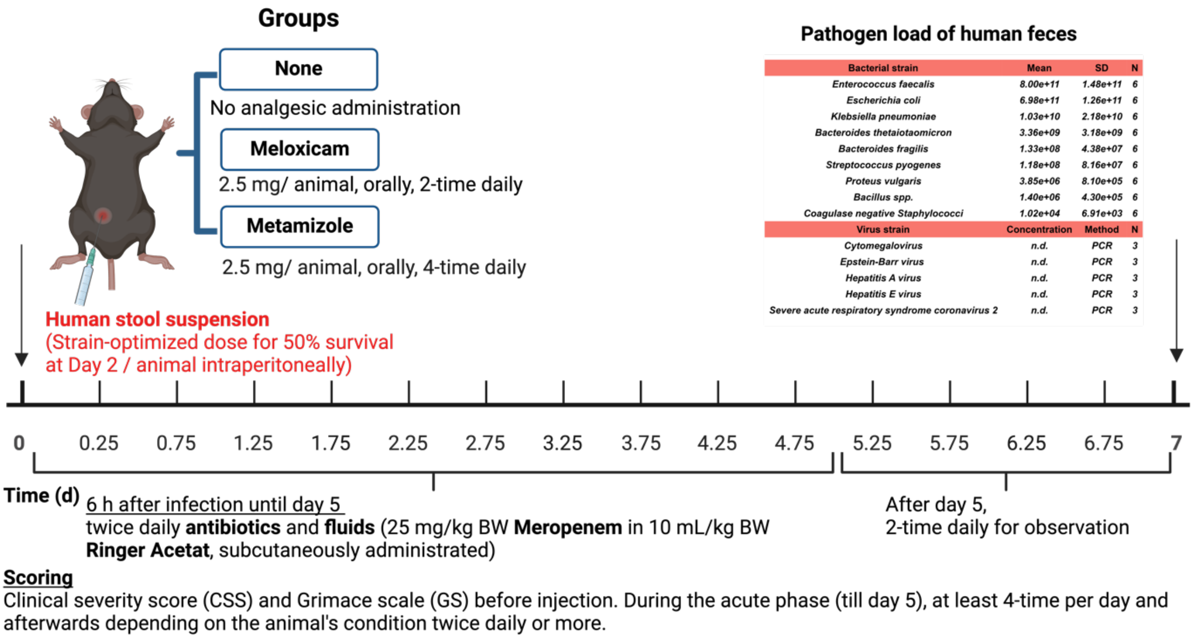

Bacterial strain	Mean	SD	N
Enterococcus faecalis	8.00e+11	1.48e+11	6
Escherichia coli	6.98e+11	1.26e+11	6
Klebsiella pneumoniae	1.03e+10	2.18e+10	6
Bacteroides thetaiotaomicron	3.36e+09	3.18e+09	6
Bacteroides fragilis	1.33e+08	4.38e+07	6
Streptococcus pyogenes	1.18e+08	8.16e+07	6
Proteus vulgaris	3.85e+06	8.10e+05	6
Bacillus spp.	1.40e+06	4.30e+05	6
Coagulase negative Staphylococci	1.02e+04	6.91e+03	6
**Virus strain**	**Concentration**	**Method**	**N**
Cytomegalovirus	n.d.	PCR	3
Epstein-Barr virus	n.d.	PCR	3
Hepatitis A virus	n.d.	PCR	3
Hepatitis E virus	n.d.	PCR	3
Severe acute respiratory syndrome coronavirus 2	n.d.	PCR	3

Supplementary Figure 1. Analgesic treatment on Peritoneal Injection and Contamination (PCI) sepsis model on C57BL/6J. All mice were divided into 3 subgroups of non-opioid analgesics, including the non-Analgesic group, the Meloxicam group, and the Metamizole group. PCI sepsis mouse model was performed via intraperitoneal injection with human stool suspension (Charge V, listed in the table for pathogen load from human feces), and mice simultaneously started analgesic administration based on a group, together with antibiotics subcutaneously applied. Each mouse was scored independently based on the Clinical Severity Score (CSS) and Grimace scale (GS) at an interval of 6 hours before day 5, while they were scored 2 times every day and stopped drug administration after day 5. If mice showed any signs of pain or CSS over 3 levels, drug treatment continued.

1.2 Supplementary Figure 2. Flow cytometry gating strategy for immune cell from tissues

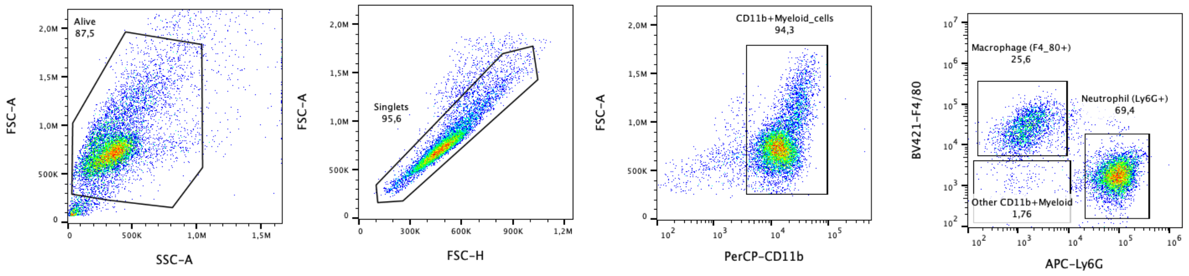

Supplementary Figure 2. Flow cytometry gating strategy for immune cell from tissues. Initially, viable cells were distinguished based on their Forward Scatter Area (FSC-A) and Side Scatter Area (SSC-A) properties. Singlet cells were then identified by comparing their Forward Scatter Area (FSC-A) and Forward Scatter Height (FSC-H). Myeloid cells were then characterized as CD11b$^+$ cells within the singlet population. These CD11b$^+$ myeloid cells were further categorized into CD11b$^+$ Ly6G$^-$ F4/80$^{+/\text{low}}$ for tissue macrophages or CD11b$^+$ Ly6G$^-$ Ly6C$^+$ for blood monocytes, CD11b$^+$ Ly6G$^+$ F4/80$^-$ neutrophils, and other CD11b$^+$ Ly6G$^-$ F4/80$^-$ myeloid cells.

1.3 Supplementary Figure 3. Colony forming units (CFUs) of lavage of septic mice with analgesic regimes

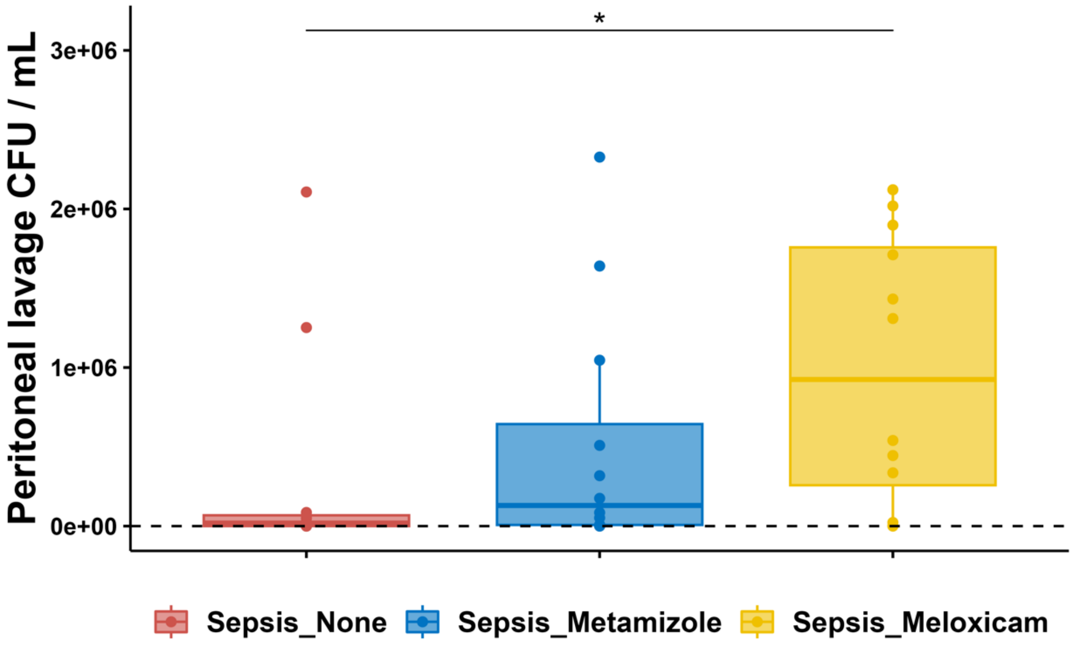

Supplementary Figure 3. Colony forming units (CFUs) of lavage of septic mice with analgesic regimes. Peritoneal lavage from four groups of mice was harvested and quantified CFUs. The median for mice from sham group is represented by a black dashline. Boxes depict the median and IQR. Dots represent individual animals, Wilcox test with HB correction was employed for P-value.

1.4 Supplementary Figure 4. Heatmap for tissue immune landscape at 24h post-sepsis treated with analgesics

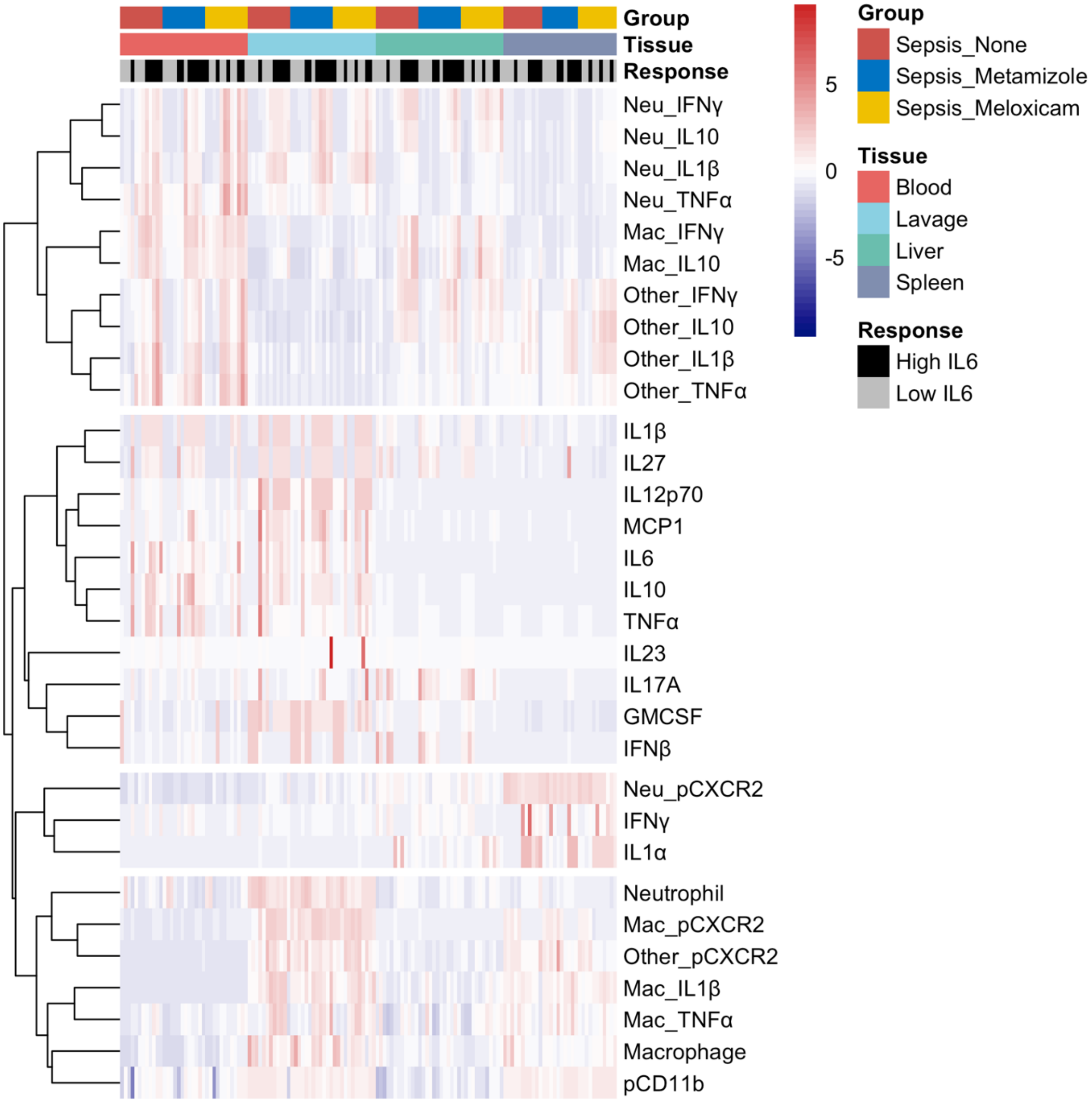

Supplementary Figure 4. Heatmap for tissue immune landscape at 24h post-sepsis treated with analgesics. Immune landscapes, including immune cells, internal synthesis, and release of inflammatory mediator, across four tissues at 24h among sepsis groups treated with three analgesic regimens, further stratified by blood IL-6 response. Name setting: CellType_internal inflammatory mediators, Neu: Neutrophils, Mac: Macrophages, Other: Other CD11b^{+} myeloid cells, CellType_pCXCR2: positive surface CXCR2 expression of cells.

Supplementary table 1. The clinical severity score (CSS) criteria and points utilized here to assess sepsis severity. This score was adopted from Gonnert et al. 2012.

**Grade**	**Quality**	**Scoring Points**	**Criteria**			
			**Spontaneous Activity**	**Reaction to external stimulus**	**Posture**	**Symptomatic**
1	no signs of illness<br><5 Points	1	• Active and strong movements (cave: consider resting phases!)	• Curious fight or flight reaction<br>• Fast movements	• Normal	• None
2	low-grade<br><9 Points	2	• Reduced activity & interrupted movements<br>• Unconditioned fur	• Reduced attentiveness<br>• Still adequate reaction	• Slightly hunched<br>• Piloerection	• Diarrhea<br>• Conjunctivitis<br>• Reddened eye/ nose region
3	mid-grade<br><13 Points	3	• Movement appears slow & tired.<br>• Difficulties to move<br>• Unconditioned fur	• Reduced reaction<br>• Delayed reaction	• Hunched<br>• Piloerection	• Loss of body weight <= 18% within 1 day
4	high-grade<br>≥13 Points	4	• Lethargic animal<br>• No movements<br>• Unconditioned fur	• No reaction	• Strong, hunched/ lethargic<br>• Piloerection present / absent	• Loss of body weight >18% within 1 day
